# Stage-Specific Proteomic Adaptations to Heme-Induced Oxidative Stress in *Aedes aegypti*: Differential Mechanisms in Larvae and Adults

**DOI:** 10.3390/ijms27020666

**Published:** 2026-01-09

**Authors:** Karla Barreto da Silva Orozimbo, Maria Aparecida Aride Bertonceli, Raquel de Souza Braga Silva, Rívea Cristina Custódio Rodrigues, Jucélia da Silva Araújo, Olga Lima Tavares Machado, Felipe Astolpho Almeida, Francisco José Alves Lemos

**Affiliations:** 1 Laboratório de Biotecnologia, Centro de Biociências e Biotecnologia, Universidade Estadual do Norte Fluminense Darcy Ribeiro, Campos dos Goytacazes, Rio de Janeiro 28013-602, RJ, Brazil; karla28orozimbo@gmail.com (K.B.d.S.O.); rivea@uenf.br (R.C.C.R.); 2 Laboratório de Química e Função de Proteínas e Peptídeos, Centro de Biociências e Biotecnologia, Universidade Estadual do Norte Fluminense Darcy Ribeiro, Campos dos Goytacazes, Rio de Janeiro 28013-602, RJ, Brazil; cidaride@hotmail.com (M.A.A.B.); raquelaghape@gmail.com (R.d.S.B.S.); jucelia@uenf.br (J.d.S.A.); olga@uenf.br (O.L.T.M.); flp_astolpho@uenf.br (F.A.A.)

**Keywords:** *Aedes aegypti*, heme toxicity, proteomics, oxidative stress

## Abstract

Heme released during blood digestion represents a major oxidative challenge for hematophagous insects, promoting the generation of reactive oxygen species (ROS) and redox imbalance. Although *Aedes aegypti* has evolved specialized mechanisms to mitigate heme toxicity, how these responses vary across developmental stages remains poorly understood. Here, we applied quantitative proteomics to compare the effects of heme exposure in larvae and adult females. In larvae, heme treatment predominantly led to downregulation of metabolic and antioxidant proteins, consistent with a shift toward energy conservation and growth regulation. Nonetheless, selective upregulation of proteins associated with mitochondrial MnSOD activity, lipid remodeling, and cytoskeletal organization indicates the engagement of complementary protective mechanisms. In contrast, adults exhibited a coordinated bioenergetic response, characterized by enrichment of mitochondrial pathways, redox-related proteins, and molecular chaperones, reflecting enhanced resilience to oxidative stress. Enrichment of cuticle-associated proteins in both stages further suggests heme-induced structural remodeling. Together, these findings demonstrate that *A. aegypti* employs divergent, stage-specific proteomic strategies to cope with heme toxicity: larvae suppress metabolic activity while maintaining structural and redox homeostasis, whereas adults reinforce mitochondrial function and proteostatic defenses. These insights advance our understanding of mosquito redox biology and highlight stage-specific vulnerabilities that may be exploited for innovative vector control strategies.

## 1. Introduction

Heme is an essential but potentially cytotoxic molecule that accumulates in the midgut of hematophagous insects after blood ingestion. Its redox-active iron center can catalyze the formation of reactive oxygen species (ROS), thereby imposing oxidative stress on cellular membranes, proteins, and organelles [1]. In mosquitoes, effective control of redox balance is therefore critical for survival and reproductive success. This is particularly relevant in *A. aegypti*, the primary vector of arboviruses such as dengue, Zika, and chikungunya [2]. Importantly, redox regulation in *A. aegypti* is also tightly linked to vector competence. Midgut redox homeostasis influences viral replication, immune signaling, and microbiota dynamics, ultimately modulating susceptibility to arboviral infection. In this context, heme-mediated redox signaling has been shown to affect global gene expression and antiviral immunity, with direct consequences for dengue virus replication and transmission efficiency [3,4,5].

Accumulating evidence highlights the central role of redox homeostasis in insect development and vectorial capacity. Heme-induced oxidative stress impacts mitochondrial function, antioxidant defenses, and proteostasis networks, which are differentially regulated across developmental stages [4,5]. Larvae experience intense growth under nutritionally constrained conditions, whereas adults must coordinate energy metabolism with reproduction and pathogen transmission. Despite this clear physiological divergence, the molecular mechanisms underlying stage-specific responses to oxidative challenges remain poorly defined. In blood-feeding females, the sudden influx of heme during digestion represents a substantial oxidative and metabolic burden that must be tightly controlled to preserve midgut integrity, support oogenesis, and maintain vector competence [3,4]. However, whether larvae and adults rely on shared or distinct heme-responsive molecular programs has not been systematically examined at the proteome-wide level.

Proteomic approaches have been widely applied in mosquitoes to identify metabolic and stress-response proteins involved in redox homeostasis and host–pathogen interactions, including in *A. aegypti* [6,7]. Comparative proteomic analyses have also revealed molecular signatures associated with insecticide resistance, highlighting differential expression of detoxification enzymes, cuticular proteins, and other stress-related factors across mosquito populations [8,9]. Together, these studies demonstrate the power of proteomics to uncover physiological adaptations to environmental and chemical stressors, yet comparative analyses addressing developmental stage-specific oxidative responses remain limited.

Here, we performed a quantitative proteomic analysis of A. aegypti larvae and adult females exposed to heme to characterize stage-specific adaptations to heme-induced oxidative stress. By integrating differential abundance analysis, functional enrichment, and literature-based comparisons, we identified distinct metabolic, structural, and stress-related strategies deployed at different life stages. These findings provide new insights into mosquito redox biology and reveal molecular vulnerabilities that may be exploited for stage-specific vector control strategies.

## 2. Results

### 2.1. Proteomic Profiling and PCA

**Figure 1:**
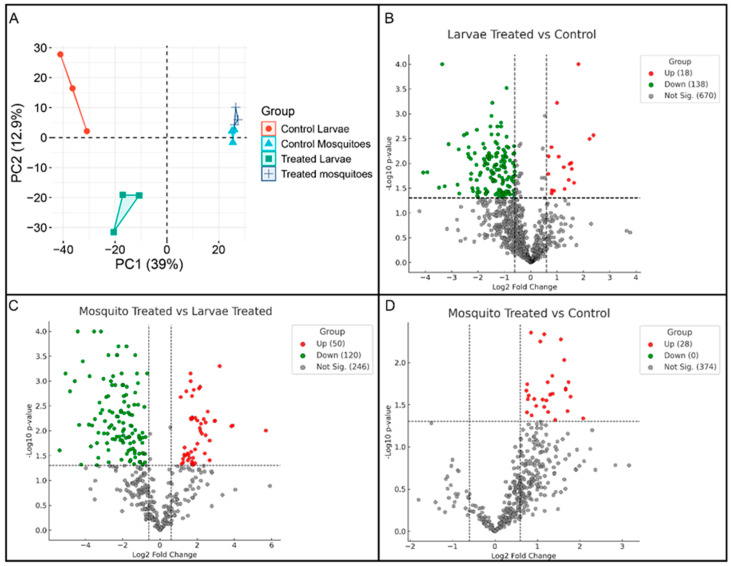
Multivariate and differential abundance analyses of *A. aegypti* proteomes under heme exposure. (**A**) Principal component analysis (PCA) of whole-body proteomic profiles from control and hemin-treated fourth-instar larvae and adult females. PC1 (39% of variance) separates samples according to developmental stage, whereas PC2 (12.9%) captures treatment- and condition-dependent variation within each stage. (**B**) Volcano plot showing differentially abundant proteins in larvae (hemin-treated vs. control). Proteins with significant differential abundance (log_2_ fold change ≥ |0.60|, *p* < 0.05) are highlighted, with upregulated proteins shown in red, downregulated proteins in green, and non-significant proteins in gray. (**C**) Volcano plot showing differentially abundant proteins in hemin-treated adult females vs. hemin-treated larvae, highlighting stage-specific proteomic adjustments under heme exposure. (**D**) Volcano plot showing differentially abundant proteins in hemin-treated vs. control adult females, illustrating the proteomic response of adult mosquitoes to heme exposure.

Proteomic analysis was conducted to evaluate the effects of heme exposure on the protein profiles of *A. aegypti* larvae and adult females. In total, 948 proteins were identified, revealing distinct proteomic signatures associated with developmental stage and treatment condition. Principal component analysis (PCA) showed that the first two components explained 39.0% and 12.9% of the total variance, respectively. Samples segregated clearly along PC1 according to developmental stage, indicating that life stage represents the major source of variability in the dataset (Figure 1A).

Heme exposure induced distinct proteomic shifts within each developmental stage, with larvae exhibiting more pronounced changes than adults. In the PCA, heme-treated larvae were positioned farther from their respective controls than treated adults, indicating greater sensitivity to heme-induced oxidative stress. Adult samples, although responsive to treatment, displayed more moderate proteomic alterations, with treated and control groups occupying adjacent but non-overlapping regions of the PCA space. Together, these patterns underscore stage-specific proteomic adaptations to heme exposure, with larvae showing heightened responsiveness (Figure 1A).

Volcano plot analyses of differentially expressed proteins indicated that heme exposure elicited stronger proteomic perturbations in larvae than in adults. In larvae, 18 proteins were upregulated and 138 were downregulated in the LarvaeT/LarvaeC comparison, whereas adults displayed 28 upregulated proteins and no downregulated proteins in the Mosq.T/Mosq.C comparison. Direct comparison between heme-treated adults and heme-treated larvae (Mosq.T/LarvaeT) identified 50 upregulated and 120 downregulated proteins. Collectively, these results indicate that larval proteomes undergo predominant downregulation in response to heme exposure, whereas adult proteomes exhibit a more limited response that is largely characterized by protein upregulation (Figure 1B–D).

### 2.2. Mfuzz Clustering and Heatmap Visualization

**Figure 2:**
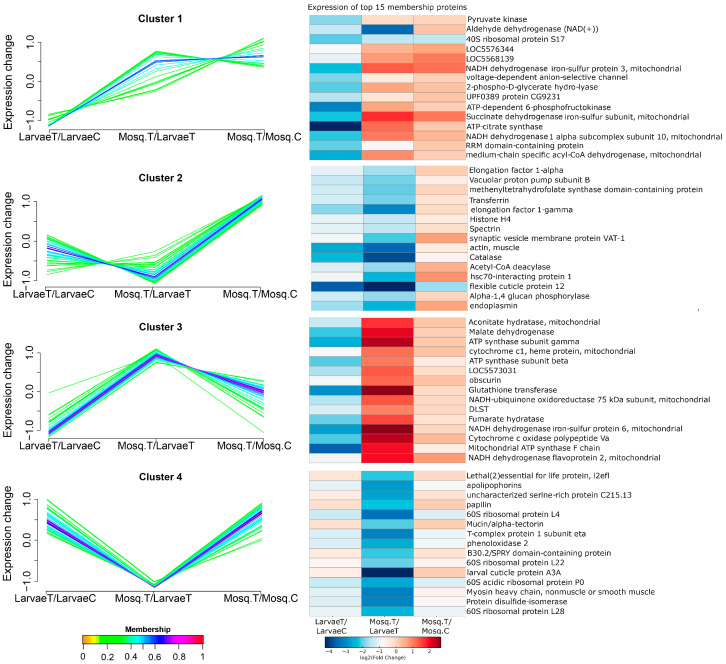
Mfuzz clustering and heatmap visualization of stage-specific proteomic trajectories under heme exposure. Proteins were grouped into four clusters based on similarities in their log_2_ fold-change profiles across the LarvaeT/LarvaeC, Mosq.T/LarvaeT, and Mosq.T/Mosq.C comparisons. Line plots (**left panels**) represent the mean expression trajectory for each cluster, while heatmaps (**right panels**) display the expression profiles of the top 15 proteins with the highest membership scores per cluster. Abbreviations: LarvaeT, hemin-treated larvae; LarvaeC, control larvae; Mosq.T, hemin-treated adults; Mosq.C, control adults.

To explore stage-specific proteomic trajectories, soft clustering was performed using Mfuzz, grouping proteins based on their log_2_ fold-change profiles across the LarvaeT/LarvaeC, Mosq.T/LarvaeT, and Mosq.T/Mosq.C comparisons. Four distinct clusters were identified, and heatmaps displaying the top 15 proteins with the highest membership scores in each cluster were generated (Figure 2).

Cluster 1 comprised proteins that were reduced in larvae and induced in adults, including glycolytic enzymes (enolase and pyruvate kinase), mitochondrial components (succinate dehydrogenase iron–sulfur subunit), and detoxification enzymes (aldehyde dehydrogenase). Cluster 2 contained proteins that were unchanged in larvae, downregulated in treated adults compared to the larvae with strong recovery in treated adults compared to control, including histones, elongation factors, and catalase.

Cluster 3 included proteins that were repressed in larvae but upregulated in treated adults, notably ATP synthase subunits, malate dehydrogenase, glutathione transferase, and muscle-related proteins. In contrast, Cluster 4 featured proteins that were upregulated in treated larvae compared to control, with strongly downregulation in treated adults compared with larvae, and recovered the upregulation in treated adults compared to control, including ribosomal proteins, cuticular proteins, and regulators of lipid metabolism. Together, these clusters reveal stage-dependent proteomic responses, with larvae exhibiting pronounced metabolic reprogramming and structural modulation, whereas adults display compensatory mitochondrial and detoxification adaptations.

### 2.3. GO Enrichment Analysis

**Figure 3:**
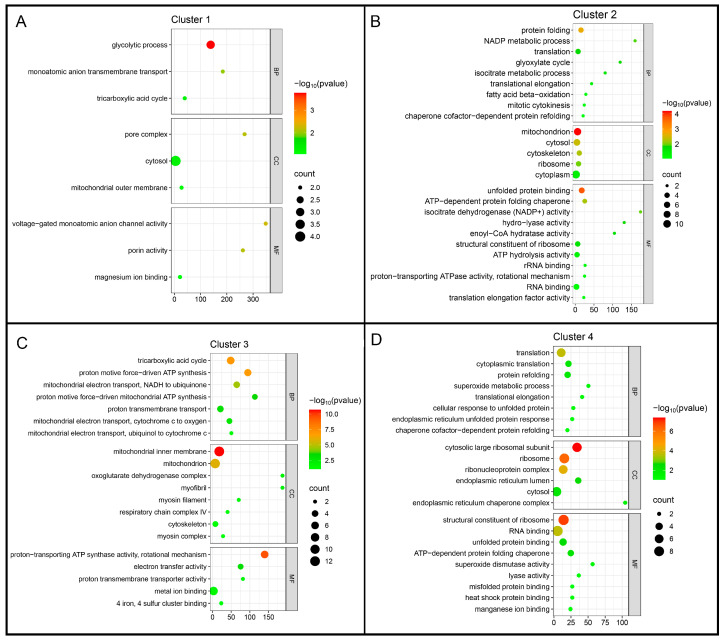
Gene Ontology (GO) enrichment analysis of Mfuzz protein clusters. Proteins from each cluster were analyzed for enrichment of Gene Ontology terms in the Biological Process (BP), Cellular Component (CC), and Molecular Function (MF) categories. (**A**) Cluster 1, enriched for pathways related to glycolysis and mitochondrial metabolism. (**B**) Cluster 2, enriched for chromatin organization and structural components. (**C**) Cluster 3, enriched for oxidative phosphorylation and ATP synthesis. (**D**) Cluster 4, enriched for ribosome biogenesis, translation, and stress-related processes, including antioxidant and protein-folding functions.

Functional characterization of the Mfuzz clusters was performed using Gene Ontology (GO) enrichment analysis across the Biological Process (BP), Cellular Component (CC), and Molecular Function (MF) categories (Figure 3).

Cluster 1 was enriched for glycolysis, the tricarboxylic acid cycle, and monoatomic anion transport. Corresponding Cellular Component (CC) terms included the cytosol, mitochondrial outer membrane, and pore complex, while Molecular Function (MF) terms were primarily associated with ion channel activity and porin functions. Cluster 2 showed enrichment for protein folding, NADP metabolism, translation, and fatty acid β-oxidation, with CC terms spanning mitochondria, cytoskeleton, ribosomes, and cytosol, and MF terms highlighting chaperone and ribosomal activities. Cluster 3 was enriched for oxidative phosphorylation and proton-motive ATP synthesis, with CC terms related to the mitochondrial inner membrane and respiratory chain complexes, and MF terms including ATP synthase and electron transfer activities. Finally, Cluster 4 encompassed translation, endoplasmic reticulum (ER) stress response, and antioxidant activity, with CC terms covering cytosolic ribosomes, the ER lumen, and ribonucleoprotein complexes, and MF terms including structural ribosomal components, RNA binding, and superoxide dismutase activity.

**Figure 4:**
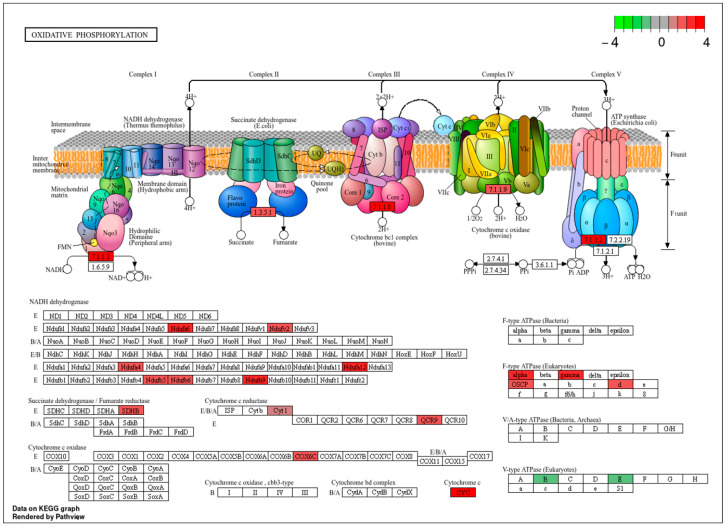
Mapping of differentially abundant proteins onto the oxidative phosphorylation pathway. KEGG-based representation of mitochondrial respiratory chain complexes I–V highlighting subunits identified in the proteomic dataset. Red boxes indicate proteins significantly upregulated in hemin-treated mosquitoes, whereas green boxes denote subunits detected in the proteomes but not differentially abundant within this pathway; no significantly downregulated proteins were observed. This pathway-level visualization summarizes the distribution of stage-specific proteomic hits within a canonical metabolic framework and represents a qualitative mapping rather than a direct measure of pathway activity, biochemical flux, or functional activation.

Taken together, these enrichment patterns indicate that heme exposure is associated with distinct, stage-specific functional programs in *A. aegypti*, rather than establishing direct causal relationships. Notably, terms related to oxidative phosphorylation were more prominent in Cluster 3. To place these mitochondrial adjustments in a pathway-level context, we mapped the differentially abundant proteins in the treated adults compared to larvae onto the oxidative phosphorylation pathway (Figure 4), highlighting the subunits detected in our dataset within this well-characterized metabolic framework.

### 2.4. Unique Proteins Define Stage- and Condition-Specific Responses

**Figure 5:**
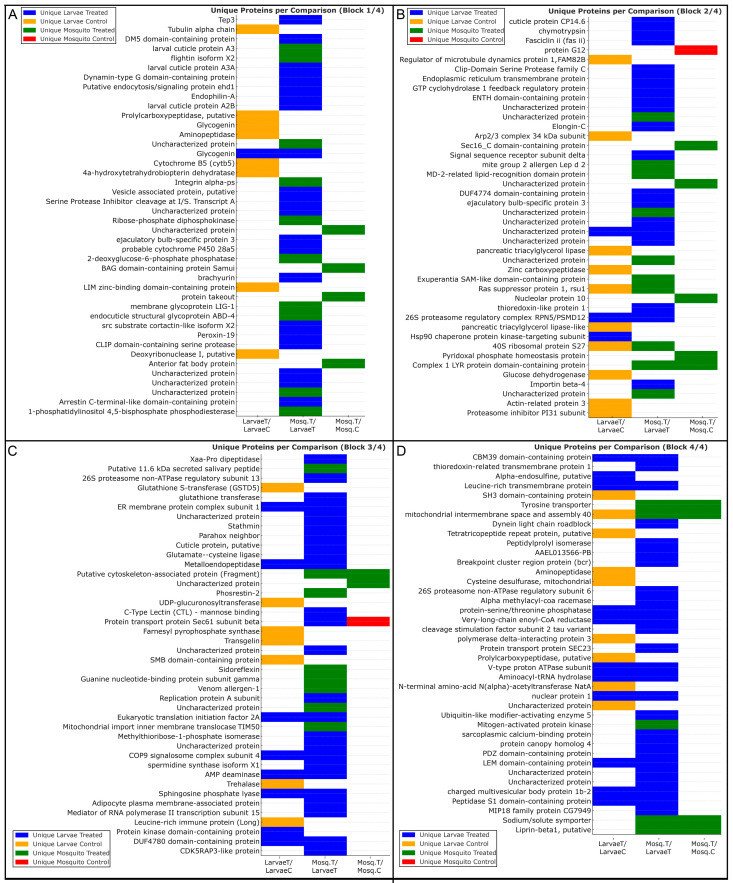
Unique proteins identified in each experimental group of *A. aegypti* under heme exposure. Proteins uniquely detected in each condition are grouped according to comparisons between larvae and adults. Each block (**A**–**D**) displays proteins exclusively identified in a specific experimental group: LarvaeT (hemin-treated larvae, blue), LarvaeC (control larvae, orange), Mosq.T (hemin-treated adults, green), or Mosq.C (control adults, red). Horizontal bars indicate the exclusive presence of each protein within the LarvaeT/LarvaeC, Mosq.T/LarvaeT, and Mosq.T/Mosq.C comparisons. Identified proteins highlight condition- and stage-specific signatures related to oxidative stress defense, detoxification, energy metabolism, muscle contractility, structural remodeling, and proteostasis, illustrating the divergence of larval and adult proteomic responses to heme-induced oxidative stress.

Analysis of unique proteins—defined as proteins consistently detected in all biological replicates of a given condition and absent from the corresponding comparator—revealed distinct condition- and stage-specific proteomic signatures (Figure 5). The distribution of these proteins across experimental groups was consistent with the patterns identified by Mfuzz clustering and Gene Ontology enrichment analyses.

LarvaeT/LarvaeC: proteins uniquely detected in heme-treated larvae were predominantly assigned to Mfuzz Clusters 1 and 4, which were enriched for Gene Ontology categories related to redox-associated processes, proteostasis, vesicular trafficking, and metabolic remodeling. These proteins included Hsp90 subunits, COP9 signalosome components, 26S proteasome particles, eIF2A, sphingosine phosphate lyase, AMP deaminase, and V-type proton ATPase subunits. In contrast, proteins uniquely detected in control larvae were mainly associated with Mfuzz Cluster 2, which was enriched for cytoskeletal organization and basal cellular functions and included α-tubulin, Arp2/3 complex components, transgelin, GSTD5, and UDP-glucuronosyltransferase.

Mosq.T/Mosq.C.: In adult females, proteins uniquely detected following heme exposure were primarily assigned to Mfuzz Cluster 3, which was enriched for Gene Ontology terms related to mitochondrial organization, oxidative phosphorylation, and ATP synthesis. This group included presequence protease, prohibitin, pyridoxal phosphate homeostasis protein, and glutathione transferase. Proteins uniquely detected in control adults were fewer and were associated with constitutive cellular processes, including sterol carrier protein 2 and Sec61 subunit beta.

Mosq.T/LarvaeT.: Direct comparison between heme-treated adults and larvae revealed distinct distributions of unique proteins across Mfuzz clusters. Proteins detected exclusively in heme-treated adults were largely assigned to Cluster 3 and were associated with mitochondrial energy metabolism and contractile-related categories, including myosin heavy chain, troponin C, ADP/ATP translocase, cytochrome *c* oxidase subunits, and Complex I components. In contrast, proteins detected exclusively in heme-treated larvae were distributed mainly across Clusters 1, 2, and 4, corresponding to Gene Ontology categories related to immune-associated processes, xenobiotic metabolism, proteostasis, and structural components, including prophenoloxidase, Tep3, clip-domain serine proteases, cytochrome P450 enzymes, proteasome subunits, eIF4C, and papilin.

Together, the distribution of unique proteins across Mfuzz clusters and associated Gene Ontology categories demonstrates distinct stage-specific proteomic profiles in response to heme exposure, as summarized in Figure 5.

## 3. Discussion

Understanding how *A. aegypti* adapts to oxidative stress across developmental stages is essential for elucidating the physiological strategies that sustain survival and vectorial capacity. Heme, an abundant pro-oxidant generated during blood digestion, catalyzes reactive oxygen species (ROS) formation and destabilizes cellular membranes, thereby imposing strong selective pressure on mosquitoes [2,3]. By integrating differential protein abundance analyses, soft clustering, and functional enrichment, this study demonstrates that larvae and adults deploy distinct, stage-specific proteomic strategies to cope with heme-induced oxidative stress. These differences reflect divergent priorities in metabolism, antioxidant defense, proteostasis, and structural maintenance across the mosquito life cycle.

### 3.1. Life-Stage Differences in Antioxidant and Detoxification Systems

Our results indicate that larvae mount a more enzyme-intensive antioxidant and detoxification response to heme exposure than adults. Proteins such as glutathione S-transferases (GSTs), glutamate–cysteine ligase, and ferritin were upregulated or uniquely detected in heme-treated larvae, underscoring a strong reliance on glutathione metabolism and iron sequestration to buffer oxidative damage. This pattern is consistent with transcriptomic evidence showing higher basal and inducible expression of detoxification genes in larval Malpighian tubules compared with adults [10].

Similar proteomic activation of antioxidant and stress-response pathways has been reported in larvae exposed to other oxidative challenges. For example, *A. aegypti* larvae treated with silver/silver chloride nanoparticles exhibited upregulation of mitochondrial and antioxidant proteins, including heat shock proteins and ATP synthase subunits [11]. Likewise, exposure to *Streptomyces* sp. KSF103 extracts induced proteins involved in metabolism and detoxification in both larval and adult stages [7]. Collectively, these studies, together with our data, support the notion that mosquito larvae primarily rely on a broad enzymatic and transporter-based antioxidant system, whereas adults employ alternative redox management strategies.

### 3.2. Heme-Driven Oxidative Stress and Antioxidant Responses

Heme toxicity arises not only from its capacity to catalyze ROS formation but also from the release of labile iron, which further amplifies oxidative stress [2,12]. In our dataset, transferrin and ferritin exhibited stage-specific regulation, indicating divergent iron-handling strategies between larvae and adults. Heme-treated larvae maintained a higher abundance of iron-binding proteins, whereas adults showed reduced transferrin levels, suggesting a diminished reliance on systemic iron transport during oxidative stress.

Larvae also displayed dynamic regulation of key antioxidant enzymes. Catalase abundance decreased under heme stress, indicating a shift away from hydrogen peroxide detoxification toward glutathione-dependent peroxidase systems [13]. In contrast, mitochondrial MnSOD was upregulated, reinforcing the first line of defense against superoxide radicals and supporting mitochondrial redox balance [14]. Adults exhibited comparatively lower abundance of catalase, SOD, and transferrin, consistent with a strategy focused on limiting intracellular ROS generation rather than broadly inducing enzymatic detoxification pathways.

Proteomic studies examining exposure to silver nanoparticles and microbial metabolites similarly reported induction of GSTs, catalase, and energy metabolism enzymes in *A. aegypti* [7,11], reinforcing the robustness of larval antioxidant responses across stress contexts. Together, these findings support a model in which larvae possess a broad, flexible enzymatic antioxidant network, whereas adults rely on more restricted and specialized redox control mechanisms.

Notably, these proteomic patterns partially contrast with transcriptomic studies of adult *A. aegypti* exposed to heme or blood meals, which reported increased expression of ferritin, GSTs, and other antioxidant genes [3,5]. Heme acts as a pleiotropic transcriptional modulator in the adult midgut, influencing redox balance, immune signaling, and dengue virus replication [4]. Large-scale RNA-seq resources, such as the Aegypti-Atlas, further demonstrate robust, time-dependent induction of iron- and redox-related transcripts following blood feeding [15].

The apparent discrepancy between whole-body proteomic profiles and gut-focused transcriptomic datasets likely reflects multiple regulatory layers, including post-transcriptional control, protein turnover, and tissue-specific localization of antioxidant proteins. Moreover, sequestration of heme by the peritrophic matrix may locally buffer oxidative stress within the midgut lumen, reducing the need for widespread systemic induction of antioxidant proteins detectable at the whole-organism proteomic level. These observations underscore the complementary nature of transcriptomic and proteomic approaches and highlight the importance of integrating tissue-specific and whole-body datasets to fully understand heme-induced stress responses in *A. aegypti*.

### 3.3. Metabolic Reprogramming and Mitochondrial Adaptation

A prominent feature across both developmental stages was the modulation of mitochondrial pathways. Heme exposure has been shown to reprogram mitochondrial gene expression and proteostasis, with consistent enrichment of oxidative phosphorylation and central metabolic pathways [4]. Proteins associated with ATP synthesis and the tricarboxylic acid cycle, including ATP synthase subunits, succinate dehydrogenase, and malate dehydrogenase, are recurrent targets of oxidative and metabolic stress [16]. These changes suggest that mitochondrial function is actively adjusted to sustain ATP production while maintaining redox homeostasis under heme-induced oxidative pressure [4,16,17].

In larvae, mitochondrial modulation occurred alongside widespread metabolic suppression, consistent with an energy-conserving strategy coupled to selective reinforcement of mitochondrial ROS detoxification. In contrast, adults displayed robust activation of mitochondrial and contractile pathways. Upregulation of citric acid cycle enzymes, electron transport chain components, and ATP synthesis machinery supports enhanced muscular performance and redox stability, aligning with studies demonstrating dynamic remodeling of flight muscle mitochondria following blood feeding [17,18]. Insect flight muscle is characterized by exceptionally rapid actomyosin cycling supported by dense mitochondrial networks, underscoring the tight integration between energy metabolism and contractile function [19].

Comparable mitochondrial adaptations have also been reported in *A. aegypti* exposed to *Streptomyces* extracts [7] and silver nanoparticles [11], indicating that shared metabolic reprogramming mechanisms support energetic and redox resilience in response to diverse stressors.

### 3.4. Structural and Proteostatic Defenses

Enrichment of cuticle-related proteins in both larvae and adults suggests that heme-induced ROS compromises barrier integrity across developmental stages. This interpretation is consistent with studies showing that ROS-generating compounds induce cuticular damage in *A. aegypti* larvae, triggering compensatory activation of chitin metabolism and cuticle repair pathways [20]. Structural reinforcement therefore appears to constitute an additional defense layer throughout mosquito development.

In adults, we additionally observed induction of stress-associated chaperones and chromatin regulators. Heat shock proteins are known to stabilize misfolded proteins and contribute to tolerance against chemical and oxidative stress, including insecticide exposure [21]. Likewise, oxidoreductin-like proteins regulated by FOXO signaling enhance mitochondrial and nuclear integrity under oxidative conditions [22,23]. Notably, adults did not exhibit the widespread protein downregulation observed in larvae, suggesting an adaptive strategy aimed at maintaining essential metabolic and protective functions under sustained oxidative load.

This stage-specific proteostatic response is further supported by studies reporting modulation of chaperones and proteasome components in mosquitoes exposed to both abiotic and biotic stressors [24,25,26], reinforcing the idea of an evolutionarily conserved mechanism for preserving cellular integrity during oxidative challenge.

### 3.5. Evolutionary Trade-Offs and Applied Perspectives

This study was designed to isolate the effects of heme as a specific oxidative stressor, independent of other blood-derived components; therefore, direct comparisons with blood-fed mosquitoes were beyond its scope. Although differential protein abundance was supported by biological replication and stringent statistical criteria, targeted validation through immunoblotting or functional assays was not performed and remains an important objective for future work.

The divergence in proteomic responses between larvae and adults underscores the central role of developmental stage in shaping oxidative stress resilience. Larvae favor metabolic suppression and extensive antioxidant investment to accommodate growth and metamorphosis, whereas adults enhance mitochondrial, proteostatic, and structural pathways to support survival and reproduction. This pattern reflects an evolutionary trade-off between larval energetic constraints and adult reproductive demands [27,28].

From an applied perspective, these stage-specific proteomic signatures reveal vulnerabilities that may be exploited for vector control. Targeting cytoskeletal or RNA-processing proteins could impair larval development, whereas inhibition of mitochondrial enzymes or chaperones may compromise adult survival and pathogen transmission [29,30,31]. Such stage-tailored strategies are consistent with emerging precision approaches to mosquito control.

Future studies should systematically explore a broader range of heme concentrations and exposure durations in both larvae and adults, integrating phenotypic, tissue-specific, and proteomic analyses to further validate and extend the stage-specific mechanisms described here.

## 4. Materials and Methods

### 4.1. Mosquito Rearing and Colony Maintenance

Mosquitoes used in this study were obtained from an *A. aegypti* Rockefeller strain colony maintained at the Biotechnology Laboratory of the Universidade Estadual do Norte Fluminense Darcy Ribeiro (UENF), Campos dos Goytacazes, RJ, Brazil. Larvae were reared in plastic trays at room temperature and fed commercial cat food. Adult mosquitoes were maintained at 27 °C, 70% relative humidity, and a 12 h:12 h light/dark photoperiod, with continuous access to a 10% sucrose solution. For egg production, 3–5-day-old females were artificially fed sheep blood, following approval by the Institutional Animal Care and Use Committee (CEUA/CBB/UENF, protocol no. 011/2023, 13 April 2023).

### 4.2. Mosquito Feeding and Larval Treatment

To establish appropriate experimental conditions for proteomic analyses, a preliminary hemin toxicity assay was conducted using fourth-instar (L4) larvae. For adult artificial feeding, sheep blood plasma supplemented with 10 mM hemin was provided via a membrane feeding system. The feeding apparatus consisted of an artificial membrane stretched over a water-jacketed glass feeder maintained at 37 °C to simulate vertebrate body temperature.

For larval treatments, L4 larvae were exposed to commercial hemin at concentrations of 5, 10, 15, or 20 mM in distilled water, while control larvae were maintained in distilled water alone. Each treatment group consisted of 90 larvae, totaling 450 individuals across all experimental conditions. Larval survival was monitored at 24, 48, and 72 h post-exposure. Kaplan–Meier survival curves were generated to determine median survival time (S50) (Supplementary Figure S1), and statistical analyses were performed using GraphPad Prism v8.0 (GraphPad Software, San Diego, CA, USA).

Based on survival outcomes (Supplementary Figure S1), exposure to 10 mM hemin for 24 h was selected as the standard condition for proteomic analyses. This condition induced robust oxidative stress without excessive mortality and is consistent with physiological heme levels encountered during blood feeding. A qualitative phenotypic assessment of treated larvae revealed no gross morphological alterations across concentrations, with treated and control larvae exhibiting comparable body shape, cuticle integrity, and segment organization (Supplementary Figure S2).

Hemin stock solutions were prepared by dissolving hemin (Sigma–Aldrich, St. Louis, MO, USA) at 100 mM in 0.1 M NaOH, followed by dilution in distilled water to the desired working concentrations. Final pH values were adjusted to 7.0–7.2 and verified using a calibrated pH meter. For larval assays, L4 larvae were transferred to plastic beakers containing 100 mL of hemin solution at a density of 20 larvae per beaker. Larvae were maintained at 27 ± 1 °C, 70–80% relative humidity, and a 12 h:12 h light/dark cycle, and received a standardized powdered diet during the 24 h exposure period.

For adult assays, 3–5-day-old females were starved for 24 h with access to water only and subsequently offered a heme-enriched artificial meal via a membrane feeder maintained at 37 °C for 1 h. Non-engorged females were removed, and fully engorged mosquitoes were maintained under standard insectary conditions (27 ± 1 °C, 70–80% relative humidity, 12 h:12 h light/dark cycle). No adult mortality was observed under these conditions. Adults used for proteomic analyses were collected 24 h after feeding.

All subsequent proteomic experiments were performed using this standardized condition in both L4 larvae and 24 h post-fed adults to enable direct comparison of stage-specific responses. Experimental groups are referred to throughout the manuscript as follows: LarvaeC (control larvae), LarvaeT (hemin-treated larvae), Mosq.C (control adults), and Mosq.T (hemin-treated adults). Comparative analyses are denoted as LarvaeT/LarvaeC, Mosq.T/LarvaeT, and Mosq.T/Mosq.C.

### 4.3. Proteomics

#### 4.3.1. Sample Preparation

Whole-body samples from larvae and adults were processed using a standardized proteomics workflow. For each sample, 100 µg of total protein was extracted, reduced with 10 mM dithiothreitol (DTT), and alkylated with 40 mM iodoacetamide (IAA). Proteins were digested overnight at 37 °C with sequencing-grade trypsin (Promega; 0.2 µg/µL) under agitation in a Thermomixer. Resulting peptides were purified using SpinColumn C18 extraction columns containing Poros R2 resin.

#### 4.3.2. Liquid Chromatography

Peptide separation was performed on an Easy-nLC 1000 system (Thermo Scientific, Waltham, MA, USA) equipped with an EASY-Spray PepMap C18 analytical column (25 cm × 75 µm, 2 µm particle size, 100 Å pores) preceded by a PepMap C18 trap column (2 cm × 75 µm, 3 µm particle size). Mobile phases consisted of:

- Phase A: 95% H_2_O, 5% acetonitrile (ACN), 0.1% formic acid (FA).
- Phase B: 95% ACN, 5% H_2_O, 0.1% FA.

Peptides were eluted at 300 nL/min using a gradient: 5% B at 0 min, 23% B at 57 min, 45% B at 77 min, and 98% B at 82 min, maintained until 90 min.

#### 4.3.3. Mass Spectrometry

Mass spectrometric analyses were performed on a Q Exactive Plus Orbitrap (Thermo Scientific) in data-dependent acquisition (DDA) mode. The instrument was calibrated on 22 January 2024, achieving mass accuracies of 0.19 ppm for precursor ions and 0.33 ppm for fragment ions. The acquisition parameters were:

- Full scan (MS1): resolution 70,000; *m*/*z* 375–1650; AGC target 3.0 × 10^6^; maximum injection time 50 ms.
- MS/MS fragmentation (HCD): top-20 most intense ions; resolution 17,500; AGC target 1.0 × 10^6^; isolation window 1.4 *m*/*z*; normalized collision energy 30; minimum ion threshold 1.0 × 10^5^; maximum injection time 100 ms.
- Dynamic exclusion: 40 s with 1.2 *m*/*z* window.

#### 4.3.4. Data Processing and Bioinformatics

Raw mass spectrometry data were processed using Proteome Discoverer v2.1 (Thermo Scientific) and searched against the *A. aegypti* UniProt reference proteome using the Sequest HT algorithm. Search parameters included a precursor mass tolerance of 10 ppm and a fragment mass tolerance of 0.1 Da. Carbamidomethylation of cysteine was specified as a fixed modification, while methionine oxidation and N-terminal acetylation were set as variable modifications. Label-free quantification was performed using MS1 ion intensities.

Data were filtered at a false discovery rate (FDR) of ≤1% at both peptide and protein levels. Only proteins identified in all three biological replicates were included in quantitative analyses, whereas proteins detected exclusively in one condition were classified as unique. Differential protein abundance was defined by a log_2_ fold change ≥ 0.60 or ≤−0.60, with statistical significance assessed using a two-tailed Student’s *t*-test (*p* < 0.05). Functional enrichment analyses were performed using OmicsBox v1.2.4 and Metascape (http://metascape.org; accessed on 5 September 2025) [32].The mass spectrometry proteomics data have been deposited to the ProteomeXchange Consortium via the PRIDE [33] partner repository with the dataset identifier PXD072226. 

## 5. Conclusions

This study provides a comprehensive proteomic characterization of *A. aegypti* larvae and adults in response to heme-induced oxidative stress. By integrating multivariate analyses, differential protein abundance profiling, soft clustering, functional enrichment, and unique protein identification, we delineated clear, stage-specific adaptive strategies. Larvae exhibited a pronounced induction of antioxidant enzymes, proteostasis regulators, and xenobiotic detoxification pathways, together with the presence of unique proteins involved in vesicular trafficking, structural remodeling, and metabolic reprogramming. In contrast, adults displayed a more selective response, characterized by reinforcement of mitochondrial protein import, energy metabolism, and structural stability, alongside a reduced reliance on broad enzymatic detoxification and transport systems.

Analysis of condition-exclusive proteins further reinforced these distinctions: larval proteomic signatures were enriched in immune effectors and metabolic enzymes, whereas adult-exclusive proteins predominantly reflected contractile machinery and mitochondrial bioenergetics. Collectively, these results demonstrate that larvae and adults do not rely on a uniform oxidative stress response but instead follow distinct proteomic trajectories that reflect their developmental priorities and physiological constraints.

Overall, our findings expand current understanding of redox biology and metabolic adaptation in *A. aegypti*, highlighting heme not only as a potent oxidative stressor but also as a signaling molecule shaping stage-specific proteomic plasticity. These proteome-wide insights provide a robust framework for future investigations into oxidative stress, mosquito development, and vector competence, and may inform the design of innovative, stage-targeted strategies for vector control.

## Data Availability

The mass spectrometry proteomics data have been deposited to the ProteomeXchange Consortium via the PRIDE partner repository with the dataset identifier PXD072226.

## Supplementary Materials

The following supporting information can be downloaded at: https://www.mdpi.com/article/10.3390/ijms27020666/s1.

## References

[1] Immenschuh S., Vijayan V., Janciauskiene S., Gueler F. (2017). Heme as a Target for Therapeutic Interventions. Front. Pharmacol..

[2] Graça-Souza A.V., Maya-Monteiro C., Paiva-Silva G.O., Braz G.R.C., Paes M.C., Sorgine M.H.F., Oliveira M.F., Oliveira P.L. (2006). Adaptations against Heme Toxicity in Blood-Feeding Arthropods. Insect Biochem. Mol. Biol..

[3] Oliveira J.H.M., Gonçalves R.L.S., Lara F.A., Dias F.A., Gandara A.C.P., Menna-Barreto R.F.S., Edwards M.C., Laurindo F.R.M., Silva-Neto M.A.C., Sorgine M.H.F. (2011). Blood Meal-Derived Heme Decreases ROS Levels in the Midgut of *Aedes aegypti* and Allows Proliferation of Intestinal Microbiota. PLoS Pathog..

[4] Bottino-Rojas V., Talyuli O.A.C., Jupatanakul N., Sim S., Dimopoulos G., Venancio T.M., Bahia A.C., Oliveira P.L., Paiva-Silva G.O. (2015). Heme Signaling Impacts Global Gene Expression, Immunity and Dengue Virus Infectivity in *Aedes aegypti*. PLoS ONE.

[5] Oliveira J.H.M., Talyuli O.A.C., Goncalves R.L.S., Paiva-Silva G.O., Sorgine M.H.F., Alvarenga P.H., Oliveira P.L. (2017). Catalase protects *Aedes aegypti* from oxidative stress and increases midgut infection prevalence of Dengue but not Zika. PLoS Neglected Trop. Dis..

[6] Martins M., Ramos L.F.C., Murillo J.R., Torres A., de Carvalho S.S., Domont G.B., de Oliveira D.M.P., Mesquita R.D., Nogueira F.C.S., Maciel-de-Freitas R. (2021). Comprehensive Quantitative Proteome Analysis of *Aedes aegypti* Identifies Proteins and Pathways Involved in *Wolbachia pipientis* and Zika Virus Interference Phenomenon. Front. Physiol..

[7] Tan K.S., Azman A.S., Hassandarvish P., Amelia-Yap Z.H., Tan T.K., Low V.L. (2023). Protein Profiling of *Aedes aegypti* Treated with *Streptomyces* sp. KSF103 Ethyl Acetate Extract Reveals Potential Insecticidal Targets and Metabolic Pathways. Int. J. Mol. Sci..

[8] Shettima A., Ishak I.H., Lau B., Abu Hasan H., Miswan N., Othman N. (2023). Quantitative Proteomics Analysis of Permethrin- and Temephos-Resistant *Aedes aegypti* Revealed Diverse Differentially Expressed Proteins Associated with Insecticide Resistance from Penang Island, Malaysia. PLoS Neglected Trop. Dis..

[9] Zhang C., Shi Q., Li T., Cheng P., Guo X., Song X., Gong M. (2021). Comparative proteomics reveals mechanisms that underlie insecticide resistance in *Culex pipiens pallens Coquillett*. PLoS Neglected Trop. Dis..

[10] Li Y., Piermarini P.M., Esquivel C.J., Drumm H.E., Schilkey F.D., Hansen I.A. (2017). RNA-Seq Comparison of Larval and Adult Malpighian Tubules of the Yellow Fever Mosquito *Aedes aegypti* Reveals Life Stage-Specific Changes in Renal Function. Front. Physiol..

[11] Chimkhan N., Thammasittirong S.N., Roytrakul S., Krobthong S., Thammasittirong A. (2022). Proteomic Response of *Aedes aegypti* Larvae to Silver/Silver Chloride Nanoparticles Synthesized Using *Bacillus thuringiensis subsp. israelensis* Metabolites. Insects.

[12] Hernandez E.P., Anisuzzaman Alim M.A., Kawada H., Kwofie K.D., Ladzekpo D., Koike Y., Inoue T., Sasaki S., Mikami F., Matsubayashi M. (2022). Ambivalent Roles of Oxidative Stress in Triangular Relationships among Arthropod Vectors, Pathogens and Hosts. Antioxidants.

[13] Day B.J. (2009). Catalase and glutathione peroxidase mimics. Biochem. Pharmacol..

[14] Candas D., Li J.J. (2014). MnSOD in oxidative stress response-potential regulation via mitochondrial protein influx. Antiox. Redox Signal.

[15] Hixson B., Bing X.L., Yang X., Bonfini A., Nagy P., Buchon N. (2022). A transcriptomic atlas of *Aedes aegypti* reveals detailed functional organization of major body parts and gut regional specializations in sugar-fed and blood-fed adult females. eLife.

[16] Voltarelli V.A., Alves de Souza R.W., Miyauchi K., Hauser C.J., Otterbein L.E. (2023). Heme: The Lord of the Iron Ring. Antioxidants.

[17] Gonçalves R.L., Machado A.C., Paiva-Silva G.O., Sorgine M.H., Momoli M.M., Oliveira J.H., Vannier-Santos M.A., Galina A., Oliveira P.L., Oliveira M.F. (2009). Blood-feeding induces reversible functional changes in flight muscle mitochondria of *Aedes aegypti* mosquito. PLoS ONE.

[18] de Carvalho S.S., Rodovalho C.M., Gaviraghi A., Mota M.B.S., Jablonka W., Rocha-Santos C., Nunes R.D., Sá-Guimarães T.D.E., Oliveira D.S., Melo A.C.A. (2021). *Aedes aegypti* post-emergence transcriptome: Unveiling the molecular basis for the hematophagic and gonotrophic capacitation. PLoS Neglected Trop. Dis..

[19] Swank D.M., Vishnudas V.K., Maughan D.W. (2006). An exceptionally fast actomyosin reaction powers insect flight muscle. Proc. Natl. Acad. Sci. USA.

[20] Bertonceli M.A.A., Oliveira A.E.A., de Souza Passos M., Vieira I.J.C., Braz-Filho R., Lemos F.J.A., Martins B.X., Façanha A.R., Pireda S., da Cunha M. (2022). Rotenoids from Clitoria fairchildiana Seeds Affect the Cellular Metabolism of Larvae of *Aedes aegypti* (Culicidae). Pestic. Biochem. Physiol..

[21] Banfi D., Bianchi T., Mastore M., Brivio M.F. (2025). The Role of Heat Shock Proteins in Insect Stress Response, Immunity, and Climate Adaptation. Insects.

[22] King B., Ikenga A., Larsen M., Sim C. (2021). Suppressed expression of oxidoreductin-like protein, Oxidor, increases follicle degeneration and decreases survival during the overwintering diapause of the mosquito *Culex pipiens*. Comp. Biochem. Physiol. A Mol. Integr. Physiol..

[23] Song J., Li Z., Zhou L., Chen X., Sew W.Q.G., Herranz H., Ye Z., Olsen J.V., Li Y., Nygaard M. (2024). FOXO-regulated OSER1 reduces oxidative stress and extends lifespan in multiple species. Nat. Commun..

[24] Weng S.C., Shiao S.H. (2020). The unfolded protein response modulates the autophagy-mediated egg production in the mosquito *Aedes aegypti*. Insect Mol. Biol..

[25] Sivan A., Shriram A.N., Muruganandam N., Thamizhmani R. (2017). Expression of heat shock proteins (HSPs) in *Aedes aegypti* (L.) and *Aedes albopictus* (Skuse) (Diptera: Culicidae) larvae in response to thermal stress. Acta Trop..

[26] Baldridge G.D., Baldridge A.S., Witthuhn B.A., Higgins L., Markowski T.W., Fallon A.M. (2014). Proteomic profiling of a robust *Wolbachia* infection in an *Aedes albopictus* mosquito cell line. Mol. Microbiol..

[27] Yan J., Kibech R., Stone C.M. (2021). Differential effects of larval and adult nutrition on female survival, fecundity, and size of the yellow fever mosquito, *Aedes aegypti*. Front. Zool..

[28] van Schoor T., Kelly E.T., Tam N., Attardo G.M. (2020). Impacts of Dietary Nutritional Composition on Larval Development and Adult Body Composition in the Yellow Fever Mosquito (*Aedes aegypti*). Insects.

[29] Airs P.M., Bartholomay L.C. (2017). RNA Interference for Mosquito and Mosquito-Borne Disease Control. Insects.

[30] Yadav M., Dahiya N., Sehrawat N. (2023). Mosquito gene targeted RNAi studies for vector control. Funct. Integr. Genom..

[31] Vanegas-Estévez T., Duque F.M., Urbina D.L., Vesga L.C., Mendez-Sanchez S.C., Duque J.E. (2024). Design and elucidation of an insecticide from natural compounds targeting mitochondrial proteins of *Aedes aegypti*. Pestic. Biochem. Physiol..

[32] Zhou Y., Zhou B., Pache L., Chang M., Khodabakhshi A.H., Tanaseichuk O., Benner C., Chanda S.K. (2019). Metascape provides a biologist-oriented resource for the analysis of systems-level datasets. Nat. Commun..

[33] Perez-Riverol Y., Bandla C., Kundu D.J., Kamatchinathan S., Bai J., Hewapathirana S., John N.S., Prakash A., Walzer M., Wang S. (2025). The PRIDE database at 20 years: 2025 update. Nucleic Acids Res..

