# Supplementary material for: Stage-Specific Proteomic Adaptations to Heme-Induced Oxidative Stress in Aedes aegypti: Differential Mechanisms in Larvae and Adults
Source: Int J Mol Sci. 2026 Jan 9;27(2):666. doi: 10.3390/ijms27020666 (PMC12840970; doi:10.3390/ijms27020666)
Supplement: Supplementary file 1 [file ijms-27-00666-s001.zip › ijms-4022070-supplementary.pdf]

## Supplementary Material

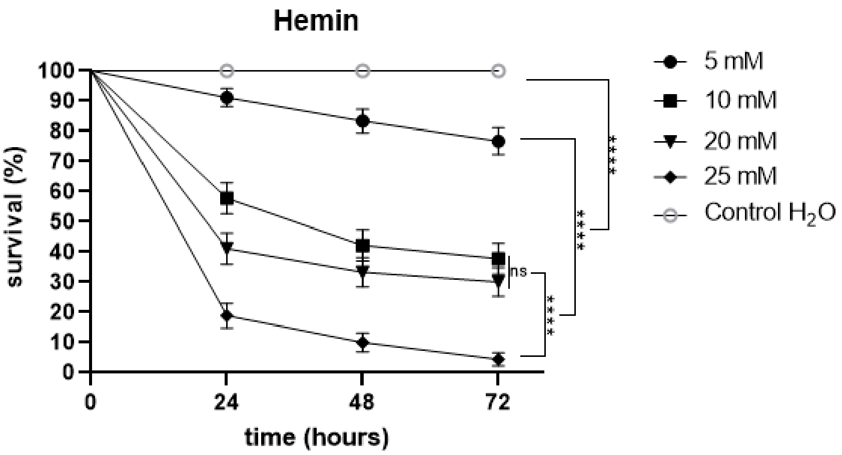


**Figura S1.** Survival of *A. aegypti* fourth-instar larvae exposed to increasing concentrations of hemin. Larvae (n = 30 per group, three independent biological replicates) were incubated in distilled water containing 0 (control), 5, 10, 20, or 25 mM hemin at 27 ± 1 ºC. Survival was assessed every 24 h over 72 h; larvae were considered alive if they responded to mechanical stimulation. Data represent mean ± SEM. ns, not significant; **P < 0.0001. Statistical analyses were performed using GraphPad Prism v8.0 (GraphPad Software,USA).

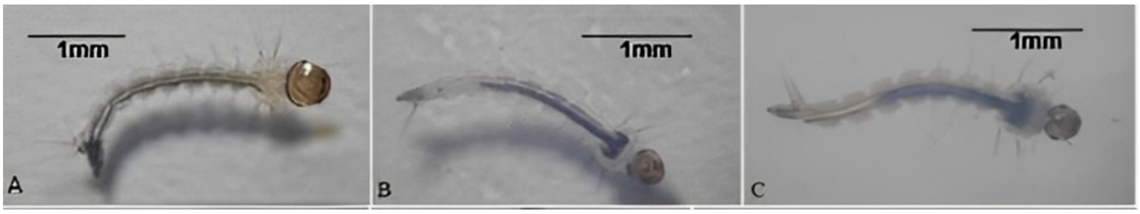


**Figure S2.** Phenotypic integrity of fourth-instar *A. aegypti* larvae exposed to hemin. Representative images of control (A) and hemin-treated larvae at 5 mM (B) and 10 mM (C) concentrations. No overt morphological alterations, deformities, or discoloration were observed. All larvae maintained similar body shape, cuticle integrity, and segment organization at the whole-body level. Scale bar: 1 mm.
